# Cholinergic white matter pathways integrity in prodromal and early manifest Lewy body disease

**DOI:** 10.1093/braincomms/fcaf421

**Published:** 2025-10-24

**Authors:** Tamir Eisenstein, Karolien Groenewald, Ludo van Hillegondsberg, Falah Al Hajraf, Tanja Zerenner, Michael A Lawton, Yoav Ben-Shlomo, Ludovica Griffanti, Michele T M Hu, Johannes C Klein

**Affiliations:** Oxford Centre for Integrative Neuroimaging, FMRIB, Nuffield Department of Clinical Neurosciences, University of Oxford, Headley Way, Headington, Oxford OX3 9DU, UK; Oxford Parkinson’s Disease Centre, Nuffield Department of Clinical Neurosciences, University of Oxford, Headley Way, Headington, Oxford OX3 9DU, UK; Oxford Centre for Integrative Neuroimaging, FMRIB, Nuffield Department of Clinical Neurosciences, University of Oxford, Headley Way, Headington, Oxford OX3 9DU, UK; Oxford Parkinson’s Disease Centre, Nuffield Department of Clinical Neurosciences, University of Oxford, Headley Way, Headington, Oxford OX3 9DU, UK; Oxford Parkinson’s Disease Centre, Nuffield Department of Clinical Neurosciences, University of Oxford, Headley Way, Headington, Oxford OX3 9DU, UK; Oxford Parkinson’s Disease Centre, Nuffield Department of Clinical Neurosciences, University of Oxford, Headley Way, Headington, Oxford OX3 9DU, UK; Department of Pharmacology & Toxicology, Faculty of Medicine, Kuwait University, Kuwait City, P. O. Box 24923, Safat 13110, Kuwait; Population Health Sciences, Bristol Medical School, University of Bristol, 5 Tyndall Ave, Bristol BS8 1UD, UK; Population Health Sciences, Bristol Medical School, University of Bristol, 5 Tyndall Ave, Bristol BS8 1UD, UK; Population Health Sciences, Bristol Medical School, University of Bristol, 5 Tyndall Ave, Bristol BS8 1UD, UK; Oxford Centre for Integrative Neuroimaging, FMRIB, Nuffield Department of Clinical Neurosciences, University of Oxford, Headley Way, Headington, Oxford OX3 9DU, UK; Oxford Parkinson’s Disease Centre, Nuffield Department of Clinical Neurosciences, University of Oxford, Headley Way, Headington, Oxford OX3 9DU, UK; Oxford Centre for Human Brain Activity, Centre for Integrative Neuroimaging, Department of Psychiatry, Warneford Hospital, University of Oxford, Oxford OX3 7JX, UK; Oxford Parkinson’s Disease Centre, Nuffield Department of Clinical Neurosciences, University of Oxford, Headley Way, Headington, Oxford OX3 9DU, UK; Oxford Centre for Integrative Neuroimaging, FMRIB, Nuffield Department of Clinical Neurosciences, University of Oxford, Headley Way, Headington, Oxford OX3 9DU, UK; Oxford Parkinson’s Disease Centre, Nuffield Department of Clinical Neurosciences, University of Oxford, Headley Way, Headington, Oxford OX3 9DU, UK

**Keywords:** Lewy body disease, Parkinson’s, RBD, cholinergic, cognition

## Abstract

Degeneration of the nucleus basalis of Meynert (NbM), the main cholinergic source to the cerebral cortex, has been demonstrated in advanced stages of Lewy body (LB) disorders. While the lateral and medial white matter pathways connecting the NbM to the cerebral cortex have been shown to be affected in LB patients with dementia, less is known regarding their vulnerability in prodromal and early manifest patients without significant cognitive impairment, and how their integrity relates to disease manifestation and progression. Here, we used diffusion MRI (dMRI) to examine whether changes in the microstructural integrity of the white matter tracts of the NbM are already evident in prodromal LB disease (namely, isolated rapid eye movement sleep behaviour disorder (iRBD), *n* = 67), and in patients with early manifest LB disease (Parkinson’s disease (PD), *n* = 73), compared to healthy controls (*n* = 53). Furthermore, we examined whether the microstructural integrity of these pathways relates to cognitive function at baseline and longitudinal follow-up, and to the risk of phenoconverting from iRBD to manifest neurodegenerative disease (PD or dementia with LBs). Lastly, we examined the potential role of the NbM as a disease epicentre in the two patient groups by spatially correlating its cortical structural connectivity profile with disease-specific (i.e., iRBD or PD) cortical atrophy patterns. We found higher microstructural integrity at baseline of both the lateral and medial pathways to be associated with better verbal fluency performance at baseline (*β* = 3.29–3.52, *P* < 0.05). Higher microstructural integrity of the medial pathway was also associated with slower decline in Montreal Cognitive Assessment (MoCA) over time (*β* = 0.05, *P* < 0.05). In addition, higher integrity of both pathways at baseline was associated with reduced future risk of phenoconversion in iRBD (HR < 0.51, *P* < 0.05). Furthermore, we found that cortical regions that are more anatomically connected to the NbM exhibited lower grey matter volumes in iRBD (*r* = −0.31, *P* < 0.05), but not PD (*r* = −0.08, *P* = 0.29), suggesting its potential role in shaping cortical pathology in iRBD. Interestingly, despite the associations observed at the subject-level, no evidence for differences in microstructural integrity of the NbM pathways was observed between patient cohorts and controls at baseline. Our findings suggest that the NbM white matter pathways have the potential to serve as non-invasive biomarkers indicating risk for clinical conversion and cortical pathology in iRBD and for baseline and longitudinal cognitive functioning in iRBD and early PD and therefore may potentially be used to stratify patients for clinical trials of disease-modifying and neuroprotective therapies.

## Teaser

Eisenstein *et al*. used diffusion MRI to examine central cholinergic white matter pathways in isolated REM sleep behaviour disorder and early PD. Higher pathways integrity is associated with slower cognitive decline and reduced risk of conversion to manifest disease, highlighting their potential as biomarkers for patient stratification and disease progression.

## Graphical Abstract

**Graphical Abstract:**
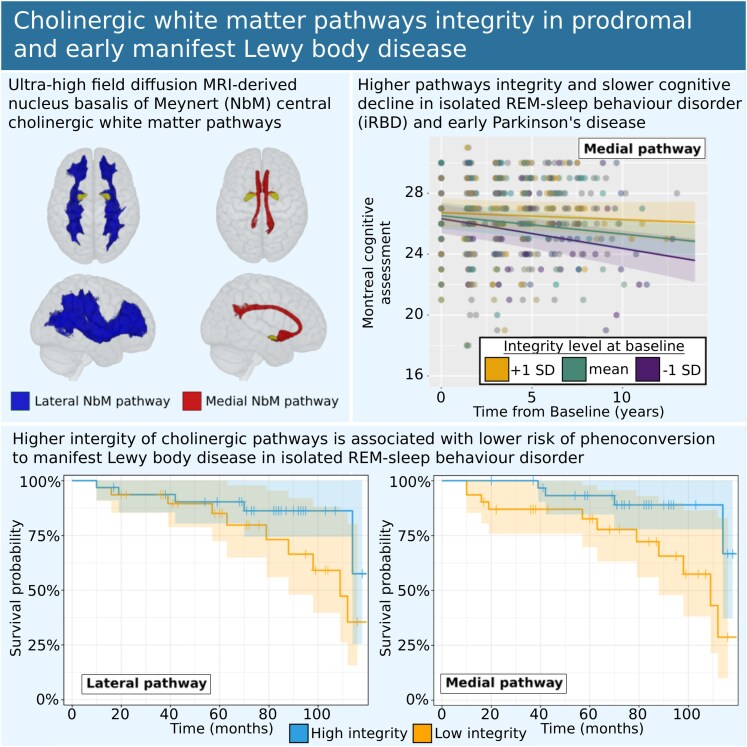

## Introduction

Cholinergic deficits have been described in Lewy body (LB) disorders such as Parkinson’s disease (PD), Parkinson’s disease dementia (PDD), and dementia with Lewy bodies (DLB), with the most pronounced cholinergic deficits found in dementia patients.^1^ While loss of central cholinergic innervation to the cerebral cortex has been proposed as a mechanism for cognitive decline and dementia in LB disorders,^2^ previous studies have shown central cholinergic degeneration and dysfunction in LB patients with and without significant cognitive impairment.^3-6^

The basal forebrain cholinergic system (BFCS) is the major source of acetylcholine (Ach) to the neocortex, hippocampus, and amygdala.^7^ BFCS neurons play key roles in shaping the activity of cortical circuits and provide important control over behavioural and cognitive processing, such as attention, visuospatial skills, and memory,^8-11^ which are prominently affected in LB disorders.^2,12^ Furthermore, cholinergic dysfunction has been proposed as a contributing mechanism to some of the non-motor features of LB disorders, such as depression, apathy, visual illusions/hallucinations, and other psychiatric symptoms.^1,13,14^

The BFCS is divided into several subregions, of which Ch4, which contains the nucleus basalis of Meynert (NbM), provides the main cholinergic innervation to the cerebral cortex. Previous studies have suggested that the NbM may be more vulnerable to LB pathology than other portions of the BFCS. Alpha-synuclein accumulation within NbM neurons was demonstrated as early as the time of nigral neuronal loss,^15^ and significant degeneration and atrophy have been reported in this region in LB disorder patients.^4,16-18^

Post-mortem studies have shown that the white matter projections from the NbM to the cortical mantle travel in two main pathways.^7,19^ A lateral pathway travels through the external capsule and uncinate fasciculus, providing cholinergic input to the frontal, parietal, occipital, and temporal cortices, while a medial pathway curves around the rostrum of the corpus callosum, enters the cingulum bundle, and innervates medial cortical regions, including the hippocampus, amygdala, cingulate, and retrosplenial cortices. Recently, diffusion-weighted magnetic resonance imaging (dMRI) has been utilized to reconstruct the trajectories of those pathways in vivo, among both patients and healthy individuals.^20,21^ Using dMRI, reduced microstructural integrity of the NbM tracts was demonstrated in patients with DLB and Alzheimer’s disease (AD).^20,22^ However, whether the white matter pathways of the NbM are already affected in early LB disorder patients without dementia, and how this may be related to the clinical manifestation of the disease, is unclear. LB disorders are characterized by a notably long and diverse prodromal stage, which can span decades. While most early prodromal markers of LB disorders, such as depression, anxiety, olfactory loss, and autonomic changes, are non-specific, a notable exception is isolated rapid eye movement (REM) sleep behaviour disorder (iRBD).^23^ iRBD is a sleep disorder characterized by the loss of muscle atonia during the REM sleep state, leading to dream enactment.^24^ It is one of the strongest clinical markers of prodromal LB disorders such as PD and DLB. Individuals with iRBD are at a high risk for a clinical diagnosis of manifest neurodegenerative disease, and previous longitudinal multicenter studies found phenoconversion rates to manifest LB disorder of 6–8% per year, exceeding all known genetic risk, and a long-term risk of phenoconversion in excess of 90%.^25-28^ Roughly equal proportions of iRBD patients progress to PD (Parkinsonism first) or DLB (dementia first).^26^ However, time to phenoconversion in iRBD can vary greatly among patients and can range from years to even decades after the onset of RBD symptoms. While iRBD has been characterized by prominent degeneration of brainstem and peripheral cholinergic nuclei,^29^ the extent to which the central cholinergic system in the brain is affected at this prodromal phase is less clear.

Therefore, the aims of the present study were four-fold. First, we investigated whether changes in the microstructural integrity of the NbM pathways are evident in early manifest and early prodromal LB disease, i.e., early PD and iRBD, respectively. We compared the disease groups to health controls. Next, we examined whether the microstructural integrity of the NbM white matter pathways is associated with baseline performance and longitudinal change of cognitive function. Then, we examined whether the microstructural integrity of the NbM pathways is associated with the risk of future phenoconversion to PD or DLB in prodromal patients with iRBD. By that, we aimed to explore the potential prognostic value of this non-invasive imaging-derived measure of cholinergic white matter integrity in future risk assessment and patients’ stratification. Lastly, as a major source of cortical innervation, we used connectivity-based epicentre analysis to examine whether the NbM could be considered a disease epicentre in iRBD and PD, i.e., a brain region whose connectivity profile may play a central role in brain-wide disease manifestation.

## Materials and methods

### Participants

The current study included data from 73 early manifest PD patients within 3 years of diagnosis, 67 participants with iRBD, and 53 healthy controls from the Oxford Parkinson’s Discovery Cohort (OPDC). The OPDC is a longitudinal observational study that aims at identifying progression markers of PD and includes a comprehensive set of clinical and MRI measurements.^30^ Participants’ demographics are summarized in Table 1. iRBD patients were verified and diagnosed with hospital-based polysomnography and were free of clinical Parkinsonism or dementia at recruitment. All participants underwent the MDS-Unified Parkinson’s Disease Rating Scale (UPDRS) parts III to assess Parkinsonian motor features, as well as the Montreal Cognitive Assessment (MoCA) to evaluate general cognitive functioning. Both iRBD and PD patients were free of significant cognitive decline at their baseline assessment. The current project was approved by the Research Ethics Board of the University of Oxford, and all participants provided written informed consent according to the Declaration of Helsinki.

**Table 1:** Demographic, cognitive, and clinical characteristics of study groups (mean ± SD are presented for continuous variables and proportions for categorical variables)

Characteristics	HC (*N* = 53)	PD (*N* = 73)	iRBD (*N* = 67)
Age (years)	66.8 (8.73)	63.9 (9.56)	66.7 (7.10)
Sex			
Female	19 (36%)	31 (42%)	7 (10%)
Male	34 (64%)	42 (58%)	60 (90%)
Education (years)	16.00 (3.57)	15.5 (3.77)	13.9 (2.72)
MoCA score (baseline)	26.8 (4.31)	27.2 (2.06)	25.9 (2.19)
Verbal fluency score (baseline)	92.9 (17.93)	80.4 (17.77)	81.5 (18.70)
UPDRS-3 total score (baseline)	1.2 (1.34)	22.0 (9.23)	4.2 (3.34)
Time from diagnosis (months)	—	10.9 (11.08)	14.5 (17.38)

### MRI acquisition

OPDC MRI data were acquired at the Oxford Centre for Clinical Magnetic Resonance Research (OCMR). A 3T Trio Siemens MRI scanner equipped with a 12-channel coil was used for data acquisition. T1-weighted scans were acquired with a 3D magnetisation prepared-rapid acquisition gradient echo (MPRAGE) sequence (192 axial slices, flip angle 8°, 1 × 1 × 1 mm^3^ voxel size, echo time/repetition time/inversion time = 4.7 ms/2040 ms/900 ms). Total acquisition time: 5:56 min. Diffusion MRI data were acquired with an EPI sequence (2 × 2 × 2 mm^3^ voxel size, echo time/repetition time/inversion time = 94 ms/9300 ms, one shell of b-values = 1000 s/mm^2^, 60 unique diffusion gradient directions, and 5 b0 images). Total acquisition time: 11:11 min.

### Diffusion MRI analysis

Diffusion MRI data preprocessing was performed using FSL (FMRIB Software Library https://fsl.fmrib.ox.ac.uk/fsl/fslwiki), and included eddy currents and head motions correction,^31^ brain extraction using FSL’s brain extraction tool (BET), and EPI distortion correction using fieldmaps. The percentage of slices identified as outliers across all participants was (0.4 ± 0.39% (mean ± SD)). Diffusion tensor models were then fitted using DTIfit, part of FSL Diffusion Toolbox. Then, tract-based spatial statistics (TBSS) workflow^32^ was utilized to non-linearly register fractional anisotropy (FA) and mean diffusivity (MD) maps into standard MNI space.

### Reconstructing the white matter pathways of the NbM

In order to create templates of the NbM white matter pathways, we utilized the preprocessed 7T dMRI data of 176 healthy young adults, which are available in the Human Connectome Project (HCP) Young Adult study (ages 22–35)^33,34^ (https://www.humanconnectome.org/study/hcp-young-adult). By using the 7T HCP young adults multi-shell dMRI data, we aimed to base the reconstruction on a high quality-high, resolution, independent dataset, with better ability to resolve crossing fibres, in order to get a more accurate spatial pattern of the NbM white matter system. In addition, by utilizing the young adult HCP dataset, we aimed to create a template of the intact cholinergic pathways, without age-related or pathology-related effects. Details of the 7T diffusion and T1w image acquisition and preprocessing protocols are provided in the HCP reference manual (https://humanconnectome.org/study/hcp-young-adult/document/1200-subjects-data-release) and the Supplementary Information.

**Figure 1:**
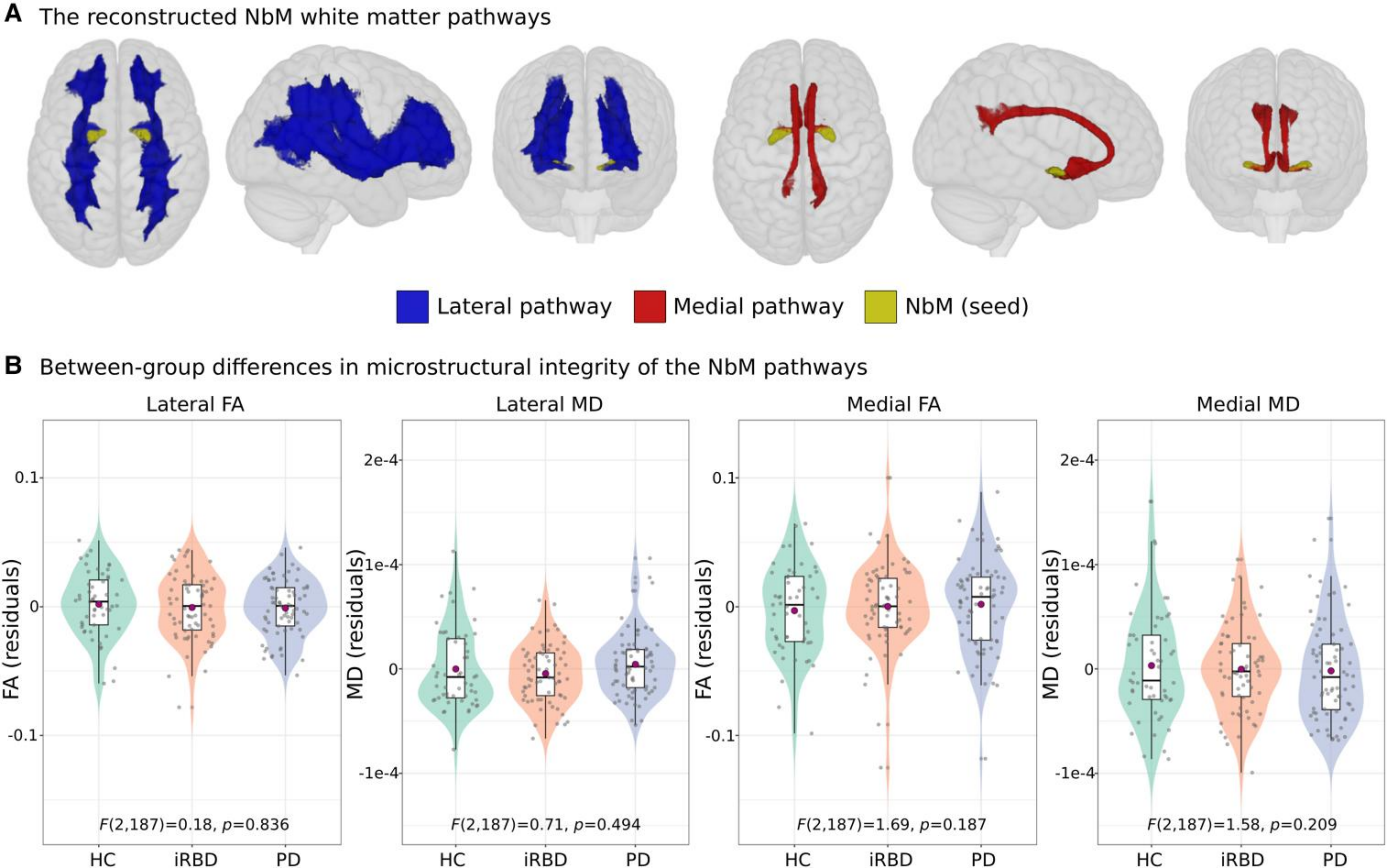
**NbM pathways integrity in prodromal and early manifest Lewy body disease**. (**A**) The reconstructed lateral and medial cholinergic pathways, seeded from the NbM, visualized using MRIcroGL (https://www.nitrc.org/projects/mricrogl/). (**B**) Analysis of covariance (*n* = 193) reviled no between-group differences in FA or MD of either the lateral or medial pathways were found between the study groups. Purple circles within boxplots represent group mean. Individual data points represent the participants in each group. FA = fractional anisotropy; HC = healthy controls; iRBD = isolated REM-sleep behaviour disorder; MD = mean diffusivity; NbM = Nucleus basalis of Meynert; PD = Parkinson’s disease.

Following preprocessing, probabilistic fibre tracking was performed on the preprocessed HCP data with FSL’s ProbtrackX2^34,35^ by generating 5000 random samples from either left or right NbM seed region-of-interest (ROI) in MNI space. The NbM seed ROIs were created using the Ch4 masks from the probabilistic cytoarchitectonic map of the Basal Forebrain (v4.2).^36,37^ A threshold at 50% was applied to probabilistic NbM masks to create the starting region for tractography, following the previously suggested threshold for realistic NbM volume estimation^38^ (Fig. 1A). Probabilistic tractography was guided by several regions of interest based on previous studies.^7,20,21^ We used binary masks of the external capsule and the cingulum from the Johns Hopkins University white matter atlas (distributed with FSL)^39^ as waypoints for the medial and lateral tracts, respectively. Stop masks localized posterior and lateral to the NbM were also used to prevent fibres from the medial pathway running posteriorly into the fornix or merging laterally into the lateral pathway. Furthermore, based on previous human post-mortem research,^19^ a retrosplenial cortical mask was used as the final stop mask for the medial pathway, while a mask covering the cortical regions reported to be innervated by the lateral pathway was used as the final stop mask for this pathway^40^ (see Supplementary Fig. 1 for visual illustration). In addition, we used several exclusion masks for each pathway, namely, a mask of the brainstem from the Harvard-Oxford atlas in FSL,^41^ mask of the anterior commissure from FSL’s XTRACT,^34,^ and masks of the contralateral hemispheres and corpus callosum. For each pathway, we also used the other pathway’s main waypoint as an exclusion mask (for example, the cingulum was used as an exclusion mask in the reconstruction of the lateral pathway).

The resulting streamline density map of each HCP participant was binarized, and group-level unilateral templates of the lateral and medial pathways were created by including all voxels that were included in at least 70% of the HCP participants’ binarized maps. This threshold was chosen based on visual inspection of the resulting pathways and previous works.^20,21^ Lastly, we added the two unilateral templates of each pathway to create bilateral medial/lateral pathway binarized templates (Fig. 1A).

### Extraction of microstructure indices

To examine the microstructural integrity of the NbM pathways, we transformed the NbM pathways templates from MNI to native space for each of the OPDC participants and extracted the average FA and MD from the diffusion tensor model’s FA and MD maps of each OPDC participant. Given that the overall (negative) correlation between the FA and MD values across all participants was high (*r* = −0.85), we performed principal component analysis (PCA) on each pathway’s FA and MD metrics to create a single microstructural integrity for that pathway represented by the resulting first PCA component (PC1). We first z-transformed each metric for each pathway and inverted the MD’s z-scores, so that both higher FA and higher MD represent higher integrity. We then ran the PCA on each pathway’s and the CC’s z-scored metrics and used the resulting first principal component (PC1) for each tract (explaining 86% and 83% of the variance for the lateral and medial pathways, respectively) as the microstructural measure for that tract in all subject-level analyses. Due to potential multicollinearity between the PC1 of the lateral and medial pathways (*r* > 0.6), we examined each pathway in a separate model and did not add both measures into the same models. To note, the extent of outlier dMRI slices detected by EDDY among the OPDC participants was weakly associated with the different diffusion metrics of the NbM pathways (*r* = 0.00–0.16, controlling for age and sex). In addition, in order to examine how specific the findings are for the NbM pathways and whether these cholinergic white matter markers merely reflect general changes in white matter integrity in RBD/PD, we also examined the average microstructural integrity across the brain’s white matter. To this end, we extracted the FA and MD values (and then computed the PC1) using a whole-brain white matter mask created with FSL FAST,^42^ from which we subtracted the two cholinergic pathways.

### NbM grey matter (GM) volume assessment

We also quantified the grey matter volume of the NbM, as previous works demonstrated its vulnerability in LB disorders,^17,18,43^ in order to assess the specificity and added value of the white matter component of this system. Values of the NbM GM volume were derived from the individual T1-weighted images using the Computational Anatomy Toolbox (CAT12, https://neuro-jena.github.io/cat/)^44,45^ implemented in the Statistical Parametric Mapping 12 (SPM12). Default CAT12 preprocessing stages were applied for processing the raw T1-weighted structural images and are further detailed in the Supplementary Information. The NbM GM volume values were then extracted from the preprocessed GM images of each participant using the Ch4 masks from the probabilistic basal forebrain cytoarchitectonic map (v4.2),^36,37^ in accordance with a previously suggested threshold for realistic estimation of the NbM volume^38^ (Fig. 1A). Total intracranial volume (TIV) was also extracted for each participant to control differences in head size. Images were visually inspected and excluded if the image quality rating (IQR) score was below 70%. The IQR is a composite measurement generated by the CAT12 pipeline, integrating image quality metrics into a single value ranging from 0 to 100 (i.e., the lower the score, the lower the image quality). The final NbM volume metric was computed as the individual residual scores of a linear regression model with NbM volume as the dependent variable and TIV and IQR as explanatory variables.

### Cognitive assessment

In addition to the global cognitive functioning evaluated with the MoCA test, participants also underwent phonemic and semantic verbal fluency tests.^46^ Both the MoCA and the verbal fluency tests were evaluated at baseline as well as during eight follow-up visits, with ∼18 months interval between each visit within the iRBD and PD groups. MoCA scores were adjusted for education level as previously suggested.^47^ The phonemic and semantic verbal fluency scores were summed within each visit to create a single total verbal fluency score measure. Supplementary Table 1 summarizes the number of participants with MoCA/verbal fluency data within each visit.

### Phenoconversion from iRBD to defined neurodegeneration

In the current study, we have focused on the two major types of phenoconversion among iRBD patients, namely to either PD or DLB, and therefore excluded participants who were diagnosed with other neurodegenerative disorders such as multiple systems atrophy. Clinical diagnosis of phenoconversion to PD/DLB in the OPDC followed standard diagnostic criteria,^48,49^ and was applied by trained neurologists by assessing the patients longitudinally with a structured series of examinations and assessments, with carer report as appropriate.

### Disease-epicenter analysis

To examine the role of the NbM as a potential origin of the disease-related structural deficits in iRBD and PD, we conducted an epicentre analysis by spatially correlating the healthy NbM-cortical structural connectivity profile (as derived from the HCP dataset) with the syndrome-specific patterns of cortical atrophy in iRBD and PD from the OPDC cohort. Regardless of its atrophy level, a region could be considered a potential epicentre if it is (i) strongly connected to other high-atrophy regions and (ii) weakly connected to low-atrophy regions.^50,51^

#### NbM-cortical structural connectivity profile

To create the healthy NbM-cortical structural connectivity profile, we again used the HCP 7T dRMI dataset and ran the probabilistic fibre tracking as detailed above using ProbtracxkX2’s network mode (–network option) to quantify the number of streamlines seeded from either the left or right NbM and 34 cortical regions in each hemisphere using the Desikan-Killiany atlas in MNI space.^52^ Streamlines seeded from the NbM to the cortical regions were classified into the lateral or medial NbM pathways as suggested and described previously^7,19,40^ (see the Supplementary Information for more details).

#### Syndrome-specific cortical atrophy profile

To create the syndrome-specific cortical atrophy profile, a w-score approach was used to account for the expected effects of age, sex, head size, and IQR on the brain morphological features.^53-55^ The w-scores are similar to Z-scores, but are adjusted for specific covariates (i.e., age, sex, head size, and IQR). Therefore, the w-score in this study represents the normal deviation of the iRBD/PD patient’s neuroimaging metric relative to the value expected in the control group (i.e., the OPDC healthy controls) for the patient’s covariates. To create w-score maps, voxel-wise linear regression analysis was first performed in the OPDC healthy control group between the preprocessed GM maps created with CAT12 and age, sex, TIV, and IQR using SPM. Then, w-score maps were computed for the iRBD and PD patients using the following formula: w-score = [(patient’s raw value) − (value expected in the control group for the patient's covariates)]/SD of the residuals in controls. From the w-score map of each patient, we extracted the mean w-scores for the 68 cortical regions in the Desikan-Killiany atlas,^52^ and then averaged the scores of each region across patients in either the iRBD or PD groups to create the syndrome-specific cortical atrophy profile. Negative w-score indicated worse/lower score compared to expected values accounting for the covariates (i.e., more atrophy/less GM volume).

#### Spatial correlation

Spearman’s rank correlation was used to correlate the spatial patterns of the healthy NbM connectivity profile with each patient group’s cortical atrophy pattern. The intrinsic spatial smoothness in two given brain images may inflate the significance of their spatial correlation, therefore, we used spin permutation tests to assess the statistical significance of these correlations.^50,56^ This methodology generates null models of overlap between cortical maps by projecting the spatial coordinates of cortical data onto the surface spheres, applying randomly sampled rotations (10 000 repetitions), and reassigning connectivity values. The original correlation coefficients are subsequently compared against the null reference distributions generated from the correlation coefficients of the spatially shuffled brain maps. We considered *P*_spin_ < 0.05 as statistically significant.

### Statistical analysis

Statistical analyses and visualizations were performed using R version 4.4.0 (https://www.r-project.org/) and the ENIGMA TOOLBOX in Python (https://enigma-toolbox.readthedocs.io/en/latest/index.html). Between-group differences in FA and MD values of the lateral and medial pathways between the three study groups were tested using analysis of covariance (ANCOVA), controlling for age, sex, and years of education. The relationship between microstructural integrity and cognitive metrics at baseline was assessed using multiple linear regression, controlling for the same covariates, as well as NbM volume (except for when testing the general white matter microstructural integrity). Both main effects and group-based interactions (pathway microstructure × disease group) were tested. If the interaction term resulted in a *P*-value > 0.05, it was removed from the final model for that measure.^57^

In order to examine the relationship between baseline microstructural integrity and longitudinal change in MoCA/verbal fluency performance over time at follow-up visits, we ran a repeated-measures linear mixed model analysis with random intercepts and random slopes using the ‘*lme4*’ package in R. Cognitive scores at each visit were the dependent variable, and participants’ ID was the random effect. Time was defined as the time in years from baseline visit to each specific visit. Each model included age, sex, years of education, and NbM volume as covariates. To examine whether NbM pathways’ microstructural integrity was related to the extent of change over time, an interaction term of Time × Microstructural integrity was added to each model.

All analyses assessing the relationship between microstructural integrity and cognition were performed on the patient participants only (controls were not included), and *P*-values were corrected for multiple tests using False Discovery Rate (FDR), separately for the cholinergic and general WM tests.^58^

To examine the association between baseline NbM tracts microstructural integrity and time to phenoconversion in the iRBD patients’ group, we conducted Cox proportional hazards regression analyses using the ‘*survival*’ package in R. Sex was not added to the models as a covariate due to the high proportion of males in this group (∼90%). In addition, phenoconversion was defined as either DLB or PD diagnosis, i.e., collapsed across both conditions, due to the number of iRBD patients phenoconverting to either PD (*n* = 12) or DLB (*n* = 5) in our cohort. Time of follow-up was defined as the time interval in months between baseline visit and either the date of conversion diagnosis among converted patients or the last follow-up visits for patients who did not convert. Only patients with at least one follow-up visit/evaluation were included in these analyses. Cases were censored when phenoconversion was diagnosed or at the last visit. The assumption of proportional hazards was verified with the Schoenfeld residuals method.

## Results

### Demographics

The main demographics and clinical features of the study groups are presented in Table 1. The group were not significantly different in age (*F*(2,190) = 2.48, *P* = 0.086) but did differ in educational level (*F*(2,190) = 6.74, *P* = 0.001) with the iRBD group having a lower number of years of education compared to either PD (*P* = 0.003, FDR-corrected) or controls (*P* = 0.007, FDR-corrected). PD and controls were not different in their educational levels (*P* = 0.441, FDR-corrected). Noting the well-known strong male predominance in iRBD, groups were significantly different in sex distribution (chi-square = 18.609, *P* < 0.001).

### NbM white matter pathways

The lateral and medial NbM tracts reconstructed using the HCP dataset are shown in Fig. 1A. Both pathways included white matter regions that are in correspondence with previous studies of the neuroanatomy of the NbM tracts.^20,21^ The lateral pathway projects from the NbM through the external capsule and uncinate fasciculus to the frontal pole and inferior frontal cortex, as well as to parietal, temporal, occipital, and insular cortices. The medial pathway projects from the NbM to the cingulum, curving around the rostrum of the corpus callosum, and continuing through the dorsal part of the cingulum to the posterior cingulate and retrosplenial cortices.

### Between-group comparison of NbM white matter pathways integrity

The ANCOVA analysis did not reveal any significant differences between the groups in FA or MD of either the lateral or the medial pathways of the NbM after controlling for age, sex, and years of education (lateral-FA: *F*(2,187) = 0.18, *P* = 0.836; medial-FA: *F*(2,187) = 1.69, *P* = 0.187; lateral-MD: *F*(2,187) = 0.71, *P* = 0.494; medial-MD: *F*(2,187) = 1.58, *P* = 0.209, see Fig. 1B). Controlling for NbM volume resulted in similar metrics (lateral-FA: *F*(2,185) = 0.14, *P* = 0.869; medial-FA: *F*(2,185) = 1.73, *P* = 0.181; lateral-MD: *F*(2,185) = 0.63, *P* = 0.536; medial-MD: *F*(2,185) = 1.61, *P* = 0.202).

The average microstructural integrity of the whole brain’s white matter also did not differ between the groups (FA: *F*(2,187) = 0.54, *P* = 0.586; MD: *F*(2,187) = 0.57, *P* = 0.567) (Supplementary Fig. 2).

This lack of group-level differences in pathways integrity contrasts with finding in the NbM grey matter volume, which was found to be significantly reduced in PD (*β* = −18.89, [95% CI −32.11, −5.67], *P* = 0.016, FDR-corrected) and also in iRBD, although not surviving FDR correction (*β* = −14.22, [95% CI −28.36, −0.07], *P* = 0.073, FDR-corrected) compared to controls (Supplementary Fig. 3). Furthermore, we did not find statistically significant associations between pathways integrity and NbM volume for either the medial pathway (*β* = −0.41 [95% CI −5.15, 4.34], *P* = 0.866, Supplementary Fig. 3) or the lateral pathway (*β* = −3.78 [95% CI −8.06, 0.50], *P* = 0.083, Supplementary Fig. 3), with no microstructure *integrity × group* interactions for each of the pathways (*P* > 0.05).

### NbM pathways, microstructural integrity, and cognitive function at baseline

#### MoCA

**Figure 2:**
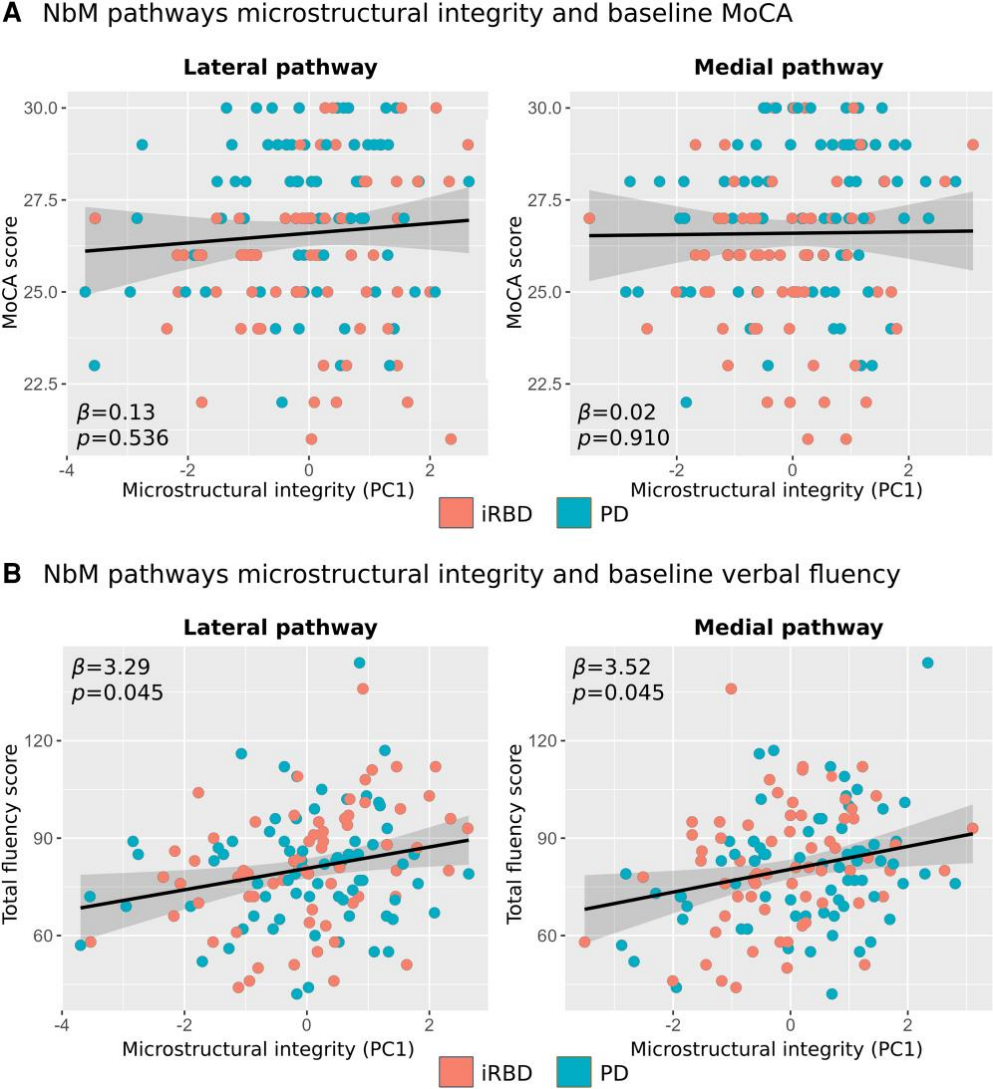
**NbM pathways integrity and cognitive function at baseline in prodromal and early manifest Lewy body disease.** (**A**) No relationship between NbM pathways microstructural integrity and MoCA performance on baseline based on linear regression analysis (*n* = 139). (**B**) Higher lateral and medial pathways integrity at baseline were positively associated with baseline verbal fluency performance based on linear regression analysis (*n* = 137). Individual data points represent individual participants in the PD group or RBD group. FA = fractional anisotropy; HC = healthy controls; iRBD = isolated REM-sleep behaviour disorder; MD = mean diffusivity; MoCA = Montreal cognitive assessment; NbM = Nucleus basalis of Meynert; PC1 = first principal component; PD = Parkinson’s disease.

We first examined whether the two patient groups differed in general cognitive functioning as reflected by the MoCA test. The iRBD group demonstrated significantly lower MoCA score at baseline visit compared to the PD patients (*β* = −1.07 [95% confidence interval (CI) −1.85, −0.28], *P* = 0.008), when age, sex, and years of education were controlled for. Weak relationships were found between baseline MoCA scores and either the lateral (*β* = 0.13 [95% CI −0.18, 0.44], *P* = 0.536) or the medial (*β* = 0.02 [95% CI −0.31, 0.35], *P* = 0.910) pathways, controlling for age, sex, years of education, group, and NbM volume (Fig. 2A). No microstructure integrity × group interactions were observed (lateral: *P* = 0.623; medial: *P* = 0.650).

Average white matter microstructural integrity was not associated with MoCA score at baseline (*β* = 0.08 [95% CI −0.23, 0.38], *P* = 0.612), and no CC integrity × group interaction was observed (*P* = 0.809) (Supplementary Fig. 4)

#### Verbal fluency

In contrast to the MoCA scores, the groups were not statistically different in verbal fluency performance (*β* = 3.05 [95% CI −3.73, 9.83], *P* = 0.376), controlling for age, sex, and education.

When examining the association between NbM pathway microstructural integrity and verbal fluency performance at baseline visit, we found higher fluency score to be associated with higher microstructural integrity of the lateral pathway (*β* = 3.29 [95% CI 0.66, 5.93], *P* = 0.045), and the medial pathway (*β* = 3.52 [95% CI 0.71, 6.33], *P* = 0.045), controlling for group, age, sex, education, and NbM volume (Fig. 2B). No microstructure integrity × group interactions were observed (lateral: *P* = 0.094; medial: *P* = 0.983).

Average white matter microstructural integrity was also found to be associated with verbal fluency score at baseline (*β* = 3.23 [95% CI 0.60, 5.85], *P* = 0.064). No CC integrity × group interaction was observed (*P* = 0.348) (Supplementary Fig. 4).

### NbM pathways, microstructural integrity, and change in cognitive function over time

#### MoCA

MoCA performance was found to significantly decline over time, controlling for age, sex, group, and education (*β* = −0.12 [95% CI −0.18, −0.06], *P* < 0.001), with no time × group interaction (*P* = 0.558).

**Figure 3:**
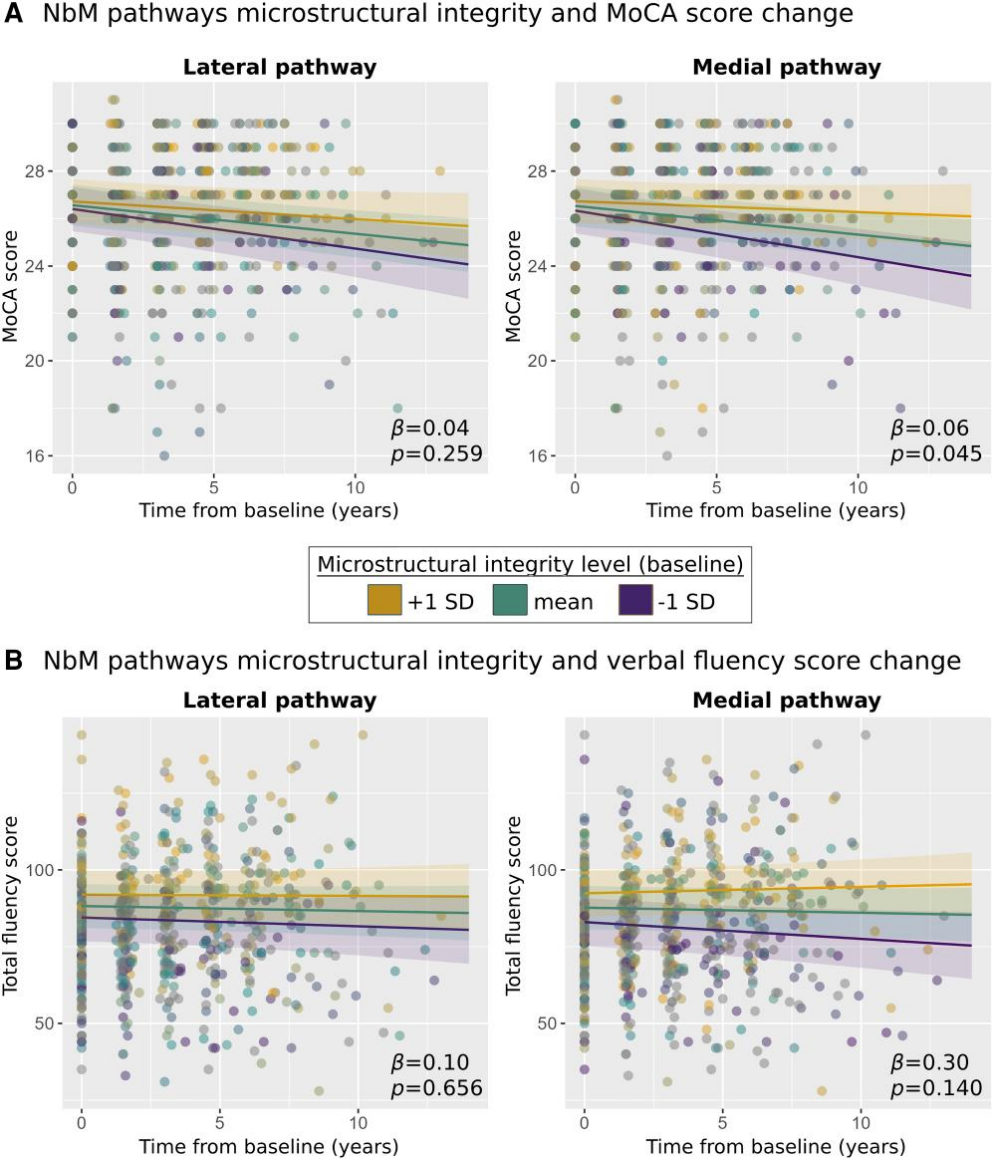
**NbM pathways integrity and change over time in cognitive function in prodromal and early manifest Lewy body disease**. (**A**) Change in MoCA score over time with a significant time × baseline microstructural integrity interaction for the medial pathway (right panel), suggesting higher medial pathway integrity at baseline is associated with slower decline in MoCA performance over time. (**B**) Change in verbal fluency score over time, as a function of the lateral and medial pathways integrity at baseline. For visualization purposes of the interaction between cognitive change over time and baseline microstructural integrity, three different slopes representing three levels of baseline microstructural integrity are presented (1 standard deviation (SD) above the mean at baseline, mean at baseline, 1 SD below the mean at baseline). The β-values represent the interaction terms’ parameter estimates in the models. The plots are based on repeated-measures linear mixed models analyses performed with longitudinal MoCA data points (*n* = 589) and longitudinal verbal fluency data points (*n* = 587) from 139 participants. MoCA = Montreal cognitive assessment; NbM = Nucleus basalis of Meynert.

Higher microstructural integrity of the medial pathway was found to be associated with slower decline in MoCA performance over time (Fig. 3A). We found significant time × microstructural integrity interaction for the medial pathway (*β* = 0.06 [95% CI 0.01, 0.11], *P* = 0.045), but not the lateral pathway (*β* = 0.04 [95% CI −0.01, 0.09], *P* = 0.259), with no evidence of a 3-way interaction effect when also adding group to the interaction term (medial: *P* = 0.860, lateral: *P* = 0.818).

No time × average WM integrity was observed (*β* = 0.04 [95% CI −0.004, 0.09], *P* = 0.162, (Supplementary Fig. 5), nor evidence of a 3-way interaction effect (*P* = 0.570).

#### Verbal fluency

Interestingly, verbal fluency performance did not significantly change over time, controlling for age, sex, group, and education (*β* = −0.17 [95% CI −0.58, 0.24], *P* = 0.425), with no time × group interaction (*P* = 0.184).

While higher microstructural integrity of the medial pathway at baseline visit was found to be associated with the extent of change in MoCA performance over time, a similar association was not observed with longitudinal change in verbal fluency for either the medial (*β* = 0.30 [95% CI −0.02, 0.63], *P* = 0.140). or lateral (*β* = 0.10 [95% CI −0.24, 0.44], *P* = 0.656) pathways. No statistically significant 3-way interaction effects with group as a moderator variable were found (medial: *P* = 0.082, lateral: *P* = 0.725).

No time × average WM integrity was observed (*β* = 0.18 [95% CI −0.13, 0.48], *P* = 0.345, (Supplementary Fig. 5), nor evidence of a 3-way interaction effect (*P* = 0.103)

### NbM pathways, microstructural integrity, and the risk of phenoconversion in iRBD

**Figure 4:**
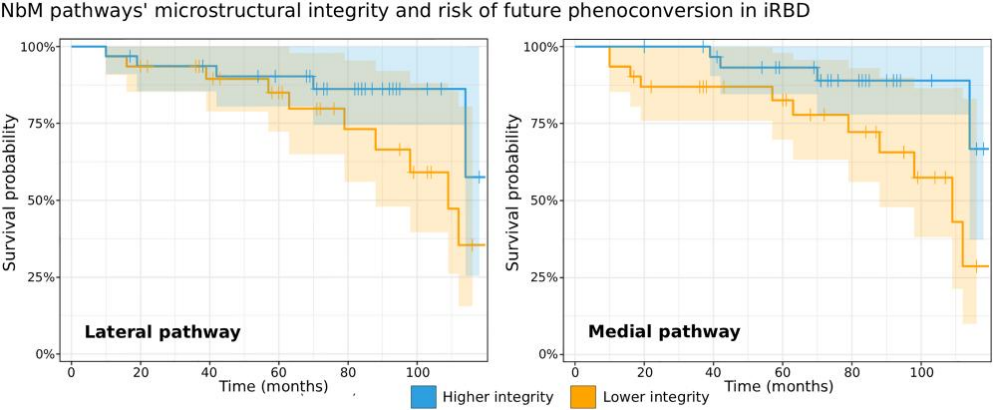
**NbM pathways integrity and risk of phenoconversion in iRBD**. For visualization purposes, we show here the survival probabilities of phenoconverting to PD/DLB in iRBD (originally assessed with Cox regression, *n* = 63), given higher or lower microstructural integrity at baseline, based on a median split of each pathway’s microstructural values adjusted for age at baseline, time from diagnosis at baseline, education, NbM volume at baseline, and UPDRS-3 and MoCA at baseline (i.e. median splitting the resulting adjusted values/the residuals). iRBD = isolated REM-sleep behaviour disorder; NbM = Nucleus basalis of Meynert.

During a mean follow-up of 71.0 ± 31.62 months, 17 patients with iRBD phenoconverted to either PD (*n* = 12) or DLB (*n* = 5). We found a 1 standard deviation increase in the lateral pathway integrity at baseline to be associated with 44% lower risk of future phenoconversion, when controlling for age and time from diagnosis at baseline visit, years of education, and NbM volume (HR = 0.56 [95% CI 0.32, 0.99], *P* = 0.045, Table 2). Adding MoCA and UPDRS-III scores at baseline as proxies of symptoms severity to further examine the added value of the lateral pathway’s microstructural integrity to the risk of phenoconversion resulted in a ∼49% reduction in risk of phenoconversion for 1 standard deviation increase (HR = 0.51 [95% CI 0.27, 0.93], *P* = 0.029, Table 2, Fig. 4).

**Table 2:** Time-to-event analysis of NbM pathways and whole WM microstructural integrity and risk of phenoconversion in iRBD patients. Hazard Ratio coefficients are per 1 standard deviation of microstructural integrity

Variable	Hazard Ratio [95% CI]	*P*-value
*Lateral pathway* (adjusted for age, time from diagnosis, education, NbM volume)	0.56 [0.32, 0.99]	**0**.**045**
*Lateral pathway* (adjusted for age, time from diagnosis, education, NbM volume, UPDRS-3, and MoCA)	0.51 [0.27, 0.93]	**0**.**029**
*Medial pathway* (adjusted for age, time from diagnosis, education, NbM volume)	0.61 [0.37, 1.03]	0.062
*Medial pathway* (adjusted for age, time from diagnosis, education, NbM volume, UPDRS-3, and MoCA)	0.31 [0.14, 0.70]	**0**.**005**
*Whole WM* (adjusted for age, time from diagnosis, education)	0.87 [0.62, 1.22]	0.416
*Whole WM* (adjusted for age, time from diagnosis, education, UPDRS-3, and MoCA)	0.85 [0.59, 1.21]	0.360

*Bold values represent statistical significance at the level of *P* < 0.05.

We also found 1 standard deviation increase in the medial pathway integrity at baseline to be associated with ∼39% lower risk of future phenoconversion, when controlling for age at baseline, time from diagnosis at baseline, years of education, and NbM volume, although consistent with chance at α-level of 0.05 (HR = 0.61 [95% CI 0.37, 1.03], *P* = 0.062, Table 2). Adding MoCA and UPDRS-III scores at baseline to the model revealed a ∼69% risk reduction for medial pathway microstructural integrity 1 standard increase (HR = 0.31 [95% CI 0.14, 0.70], *P* = 0.005, Table 2, Fig. 4). Associations between the pathways’ integrity and the UPDRS-III scores are presented in the Supplementary Fig. 6.

In contrast, while 1 standard deviation increase in general WM microstructural integrity was associated with ∼13% reduced risk of phenoconversion, when controlling for age at baseline, time from diagnosis at baseline, and years of education (HR = 0.87 [95% CI 0.62, 1.22], *P* = 0.416), and ∼15% reduced risk when also controlling for MoCA and UPDRS-III scores (HR = 0.85 [95% CI 0.59, 1.21], *P* = 0.360), it was consistent with chance at α-level of 0.05 in both models (Table 2).

### NbM as a disease epicentre in iRBD and PD

**Figure 5:**
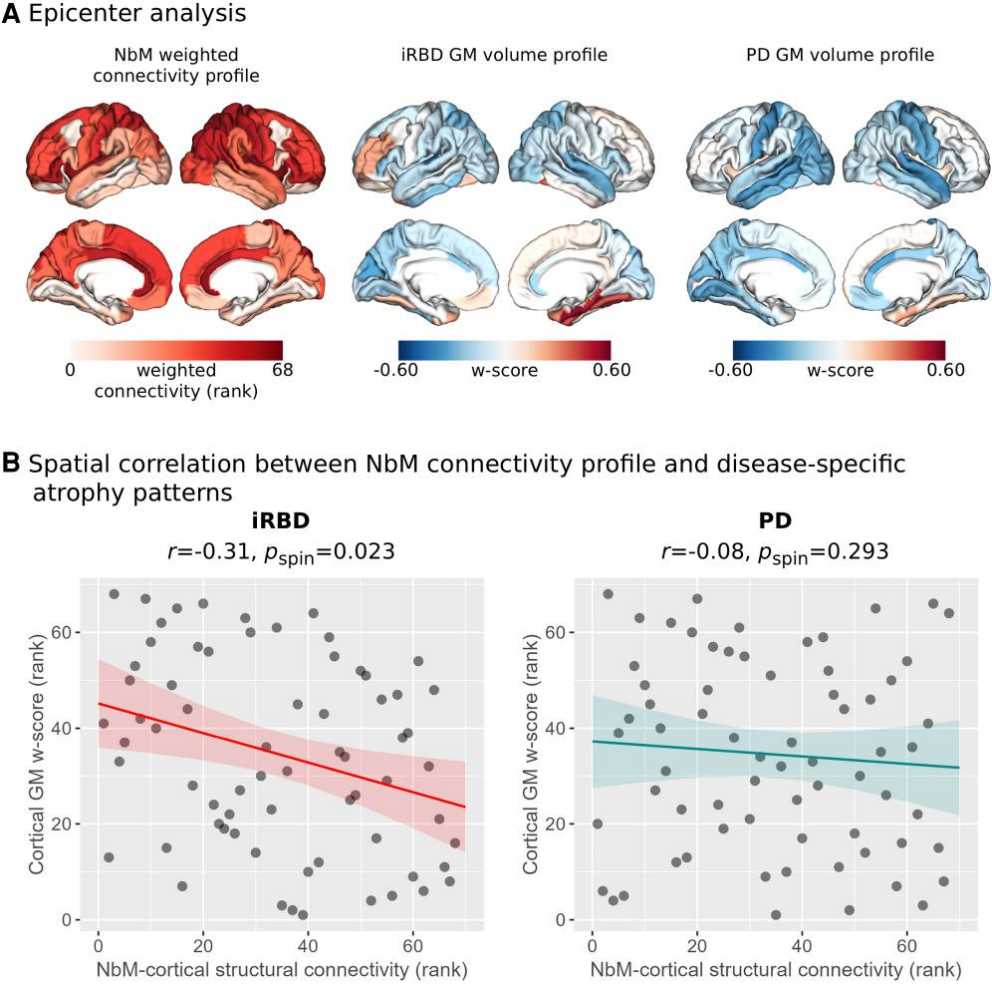
**NbM as a potential syndrome-specific epicenter in prodromal Lewy body disease**. (**A**) Disease epicenter analysis scheme, based on normative NbM-cortical structural connectivity profile derived from the HCP dataset (left panel, regions with greater weighted connectivity are presented in darker red), and syndrome-specific cortical GM volume patterns in iRBD (middle panel) and PD (right panel), expressed as w-scores relative to the healthy controls in the OPDC cohort. (**B**) Spearman correlation analysis reviled association between NbM connectivity profile and atrophy patterns in iRBD (left panel, *n* = 67), but not PD (right panel, *n* = 73), suggesting its potential role as origin of cortical structural deficits in this prodromal syndrome. Individual data points in each plot represent the participants in that group. GM = grey matter; iRBD = isolated REM-sleep behaviour disorder; NbM = Nucleus basalis of Meynert; PD = Parkinson’s disease.

Lastly, we aimed to examine the potential role of the NbM as a disease epicentre in iRBD and PD by examining the spatial relationship between the structural connectivity profile of the NbM and the cortical atrophy patterns of both patient groups. Both iRBD and PD patients demonstrated a general anterior-to-posterior pattern of cortical volume differences compared to controls, with negative w-scores (i.e. lower volume/higher atrophy) found predominantly in posterior cortices (Fig. 5A). We found a significant negative spatial correlation between the cortical atrophy profile and the healthy HCP-derived structural connectivity profile of the NbM in the iRBD group (*r* = −0.31, *p_spin_* = 0.023), suggesting that cortical regions that are more anatomically connected to the NbM also demonstrated a higher degree of atrophy in this disease group (Fig. 5B). We did not observe a spatial relationship between atrophy patterns and NbM connectivity patterns in the PD group (*r* = −0.08, *P* = 0.293) (Fig. 5B).

## Discussion

Cellular and grey matter degeneration of the NbM has been demonstrated in PD patients with and without dementia,^4,43,59^ and was recently suggested to be evident already in patients with iRBD.^43,60^ However, despite the early suggested involvement of NbM pathology in LB disorders,^15^ there is less evidence regarding the white matter pathways originating from the NbM in prodromal and manifest LB disorder patients without cognitive decline. The present study aimed to investigate the microstructural integrity of NbM white matter pathways in patients with iRBD and patient with early manifest PD without dementia, to examine whether the integrity of those pathways is associated with cognitive performance and disease progression (i.e. longitudinal change in cognitive function and phenoconversion), and whether the NbM, through its structural connections with the cerebral cortex, could be considered a disease epicenter in those patient groups, potentially shaping cortical structural deficits.

The NbM cholinergic system has been implicated in cognitive dysfunction in LB disorders, patients with an emphasis on attentional, visuospatial, and memory processing.^2,9,12^ In the current study, we found a relationship between the microstructure integrity of the NbM pathways and baseline and longitudinal cognitive function. Specifically, significant associations were observed between higher microstructural integrity and baseline verbal fluency score, as well as slower decline in global cognitive functioning as reflected by the change in MoCA performance over time. Interestingly, no significant relationship was observed with MoCA score at baseline, which could have resulted from the relatively limited range of patients’ MoCA scores in the current study, as they do not present with significant cognitive deficits. However, increased microstructural integrity of the medial pathway at baseline was indeed associated with milder decline in MoCA over time. Previous studies have demonstrated associations between the NbM pathways integrity and better performance across various cognitive tests in patients and healthy populations. For instance, Schumacher and colleagues found lateral NbM tract integrity to be associated with the Mini Mental State Examination (MMSE) test for global cognition, and to predict performance on a choice reaction time test across patients with MCI and manifest AD and DLB.^20^ Nemy and colleagues found a relationship between the lateral NbM tract and delayed recall, and between the medial NbM tract and reaction time test and delayed recall in cognitively intact adults.^21^ In another study, Nemy and colleagues found an association between lateral and medial tracts and tests of attention and memory in MCI and AD patients, as well as between the lateral pathway and attention in individuals with normal cognition.^22^ Interestingly, Hepp and colleagues did not find significant association between NbM tracts integrity and performance on the Cambridge Cognitive Examination-Revised test battery (CAMCOG) among PD patients.^61^ Most of those tests in which performance was found to be associated with NbM pathways integrity rely on multiple and higher cognitive processes for successful performance, including attention and memory, which have been particularly linked with the function of the central cholinergic system. Performance on the verbal fluency test, which was found to be related to the NbM pathways integrity in the current study, has also been associated with multilevel cognitive processes such as attention, working memory, semantic memory, executive functioning and language,^46,62^ which in turn is in line with the role of the central cholinergic system in modifying the activity of multiple functional cortical circuits. Furthermore, it is possible that an association between the general MoCA test and cholinergic markers may be more apparent as the disease progresses, especially among early manifest patients without cognitive deficits at their baseline assessment.

In addition to cognitive function, one of our most meaningful findings was the potential predictive value of the microstructural integrity of the NbM pathways in terms of disease progression in the prodromal phase of LB disease (namely, in iRBD patients). Specifically, lower pathway integrity was strongly associated with increased risk of phenoconversion in iRBD patients. This predictive relationship persisted even after rigorous control for disease severity symptoms such as UPDRS-3 and general cognitive functioning, highlighting these pathways’ potential as an independent biomarker for patient risk stratification—particularly valuable for participant selection in clinical trials of disease-modifying and neuroprotective therapies. While our sample size precluded differentiation between phenoconversion to PD versus DLB, this limitation may be less consequential than initially apparent. Even large multi-center collaborations have struggled to establish clear distinctions between these conversion pathways.^26^ This difficulty likely reflects the fundamental nature of these conditions as PD and DLB share similarities in clinical presentation, and iRBD patients convert to either condition in roughly similar proportions.^26^ Indeed, growing evidence suggests these conditions may not be distinct neuropathological entities, but rather different manifestations along a spectrum of the same underlying disease process.^26^ Our findings align with and extend previous research on the NbM tracts’ predictive value in neurodegenerative progression. Notably, Schumacher and colleagues demonstrated that reduced microstructural integrity of the NbM pathways predicted a higher risk of dementia onset in MCI patients.^20^ Furthermore, the observation of different associations for the cholinergic pathways and general white matter with longitudinal cognitive function and risk of phenoconversion supports a specific effect of the cholinergic system modulating risk in these patient groups. Together, these results strengthen the emerging role of NbM pathways integrity as a valuable prognostic marker across different stages of LB pathology. To further support the predictive role that the NbM white matter system may play in iRBD, we also found this region, through its cortical white matter connections, to be associated with the degree of cortical atrophy in iRBD patients, but not in PD. It is possible that the cholinergic system may play a more central role in iRBD compared to early manifest PD without cognitive decline, given the high proportion of dementia conversion in this prodromal subtype of LB disorders^26^ and the well-documented vulnerability of this system in LB dementia.^9^

Due to the spatial resolution constraints of MRI, we were not able to specifically reconstruct the specific axonal fibres directly originating from the cellular bodies of the NbM neurons,

Interestingly, while we successfully reconstructed the lateral and medial NbM white matter pathways as previously described,^7,19-21^ and despite the well-documented vulnerability of NbM neurons in LB disorders and subject-level associations we observed, we did not find significant differences in the microstructural integrity of either the lateral or medial pathways between the patients’ groups and controls. These findings in prodromal and manifest LB disorders without cognitive decline are partially in contrast to a previous study by Schumacher and colleagues,^20^ where significant differences in the lateral, but not the medial pathway’s microstructural integrity were found in prodromal DLB (i.e., probable MCI with Lewy bodies) and manifest DLB patients. However, those prodromal DLB patients (in contrast to the iRBD cohort in the current study) were already presenting with objective cognitive deficits, and previous works have highlighted more severe reductions in cholinergic nerve terminal integrity in LB dementia, either PD dementia (PDD) or DLB, compared to LB disorders without dementia.^3,59^ Furthermore, Hepp and colleagues^61^ have also not found differences in the MD of NbM tracts between PD patients, either with or without hallucinations, and controls. Interestingly, the lack of group-level deficits in the NbM white matter system, despite observed reductions in NbM grey matter volume in both patient groups compared to controls, advocates against a ‘dying-back’ mechanism of neurodegeneration in the NbM cholinergic system. According to this mechanistic hypothesis, which was previously suggested to manifest in monoaminergic neurotransmitter systems in PD,^63,64^ the pathological process may propagate in a ‘top-down’ manner, such that axonal and terminal pathology precede cell body degeneration. However, the lack of evidence for such a process in the current study should be interpreted with caution. The two MR modalities used in the current study (i.e., volumetric and diffusion) derive their signal from different biophysical properties of the underlying tissue and therefore may differ in their sensitivity to the underlying pathological features across different tissue types (e.g., grey versus white matter). For instance, it is possible that standard diffusion metrics derived from diffusion tensor imaging (DTI), such as FA and MD, are not sensitive enough to detect cholinergic white matter loss in prodromal and early manifest patients without cognitive decline, where the expected deficits could be more subtle or less robust at the group level. Hence, in patients without objective cognitive impairment and at an earlier disease stage (such as in the current study), milder degeneration of the NbM projections may not be detected by standard dMRI metrics. More advanced dMRI sequences such as multi-shell acquisitions, may prove helpful for future studies but were not available in our patient groups. These may enable modelling sub-components of the microstructural environment within each voxel which may confound the results of traditional DTI metrics due to partial volume effects, limited resolution of different fibre populations, and may also enable more meaningful mechanistic biological insights.^65,66^

## Limitations

The NbM white matter system is composed not only of the white matter tracts but also their synaptic terminals innervating the target cortical neurons. This component of the system has been associated with clinical and cognitive measures in LB disorder patients before, and was shown to be more severe in patients with dementia, either PD or DLB, compared to LB disorders without cognitive decline.^3,17,67^ However, while we were not able to examine synaptic terminals activity/integrity in the current study, we have accounted for the NbM grey matter volume in the analyses, which follows previous works showing an independent effect for the NbM white matter pathways.^20-22^

Despite iRBD and PD representing distinct phases on the LB disease continuum—potentially separated by more than a decade—we did not detect statistically significant group-based interactions between these cohorts regarding the relationship between NbM tract integrity and clinical or cognitive measures. However, this absence of significant interactions should be interpreted with caution. Statistical detection of interaction effects typically requires substantially larger sample sizes compared to main effects, suggesting our study may have been underpowered to identify such group-based differences, even though we successfully detected significant main effects.^68^ Additionally, our relatively modest number of iRBD-to-manifest disease converters underscores the need for validation studies with larger converter cohorts to strengthen and extend our findings.

Lastly, in the current study, we used state-of-the-art high-resolution 7T dMRI data to reconstruct the two NBM pathways that have been described previously in post-mortem and in vivo neuroimaging studies. However, despite the spatial similarity of the reconstructed tracts in the current and previous dMRI studies with the NbM pathways identified in human post-mortem works,^7,19^ the contribution of the actual cholinergic fibres to these pathways cannot be isolated from the other fibre systems with current in-vivo dMRI methodology.

## Conclusions

Our study reveals that while NbM white matter pathways’ microstructural integrity does not significantly differ between controls and early-stage patients (iRBD or early PD without cognitive decline), the degree of pathway integrity holds important clinical significance. Specifically, pathway integrity correlates meaningfully with both baseline and longitudinal cognitive performance in these patient populations. More importantly, our findings establish NbM pathway microstructural integrity as a promising non-invasive imaging biomarker for predicting disease progression risk and potential phenoconversion to manifest disease in iRBD patients and suggest an important potential role of the NbM in shaping disease-related structural cortical deficits. These results point towards a valuable tool for patient stratification in clinical trials focused on disease-modifying and neuroprotective therapies. To strengthen these findings and establish their clinical utility, future research should focus on validating these results in larger cohorts, particularly with expanded samples of phenoconverters.

## Supplementary Material

fcaf421_Supplementary_Data

## Data Availability

OPDC data are available upon reasonable request. Qualified investigators seeking access to de-identified participant data relating to the OPDC may submit their request by means of a formal application to the Oxford Parkinson’s Research Centre (OPDC) Data Access Committee. The application form, protocol, and terms and conditions may be found at https://data.dpuk.ukserp.ac.uk/cohortdirectory/Item?fingerPrintID=OPDC%20Discovery and https://www.dpag.ox.ac.uk/opdc/research/external-collaborations. The lateral and medial pathways’ masks used in the current study can be found and downloaded at https://github.com/TamirEisenstein/Cholinergic_WM_LewyBody. The code for the epicenter analysis used in the current study was based on the code found at https://enigma-toolbox.readthedocs.io/en/latest/pages/07.epicenter/index.html.
