# Supplementary material for: Cholinergic white matter pathways integrity in prodromal and early manifest Lewy body disease
Source: Brain Commun. 2025 Oct 24;7(6):fcaf421. doi: 10.1093/braincomms/fcaf421 (PMC12624394; doi:10.1093/braincomms/fcaf421)
Supplement: fcaf421_Supplementary_Data [file fcaf421_supplementary_data.pdf]

Supplementary Information

Reconstructing the white matter pathways of the NbM

The DWI scans in the HCP dataset were acquired with the following main parameters: 1.05mm isotropic voxels, 132 transversal slices acquired in interleaved order without a gap, phase encoding applied along the anterior–posterior direction, two shells with b-values = 1000, 2000 s/mm^2, repetition time/echo time = 7000/71.2 ms, 65 unique diffusion gradient directions and 6 b0 images were obtained for each phase encoding direction pair (AP and PA pairs). The total scanning time for the dMRI protocol was about 40 min.

The dMRI preprocessing was performed as described by Glasser and colleagues[1], based on the updated diffusion pipeline (v3.19.0), including basic preprocessing, distortion correction, eddy current correction, motion correction, gradient nonlinearity correction, and registration of the mean b0 image to native T1w images with FSL's FLIRT BBR + bbregister and transformation of diffusion images. The brain mask of each subject is based on FreeSurfer segmentation. Nonlinear transformations to standard space (MNI152) were obtained using the respective T1-weighted images using FSL's FNIRT[2,3]. Next, estimation of the diffusion parameters was carried out using FSL's BedpostX in a ball-and-sticks model for each voxel[4], considering three fibres modelled per voxel.

NbM grey matter (GM) volume assessment

The T1-weighted images were resampled, corrected for bias field inhomogeneities, affine-registered, and segmented into brain tissue classes (i.e., grey matter (GM), white matter (WM), and cerebrospinal fluid (CSF)). Further preprocessing included local tissue intensity transformation, partial volume estimation, and spatial normalization to standard MNI space using DARTEL. The spatially normalized images were then "modulated" by multiplying the voxel values with the Jacobian determinant (i.e., linear and non-linear components) derived from the spatial normalization. This allows the extraction of the absolute amount of tissue (e.g. "volume" of grey matter)[5]. GM volumes for further analyses were extracted from the final preprocessed GM volume maps, i.e., the modulated, normalized, partial volume corrected GM images of each participant.

Supplementary Table 1. MoCA and verbal fluency data availability for the patient groups at baseline and follow-up visits

Characteristics	PD	iRBD
Baseline visit	MoCA: N=73<br><br>Verbal fluency: N=72	MoCA: N=67<br><br>Verbal fluency: N=67
Visit 2	MoCA: N=69<br><br>Verbal fluency: N=69	MoCA: N=60<br><br>Verbal fluency: N=61
Visit 3	MoCA: N=59<br><br>Verbal fluency: N=59	MoCA: N=51<br><br>Verbal fluency: N=50
Visit 4	MoCA: N=46<br><br>Verbal fluency: N=46	MoCA: N=42<br><br>Verbal fluency: N=42
Visit 5	MoCA: N=31<br><br>Verbal fluency: N=31	MoCA: N=33<br><br>Verbal fluency: N=33
Visit 6	MoCA: N=18<br><br>Verbal fluency: N=18	MoCA: N=26<br><br>Verbal fluency: N=26
Visit 7	MoCA: N=11<br><br>Verbal fluency: N=11	MoCA: N=11<br><br>Verbal fluency: N=11
Visit 8	MoCA: N=3<br><br>Verbal fluency: N=3	MoCA: N=6<br><br>Verbal fluency: N=6
Visit 9	MoCA: N=3	MoCA: N=2

Characteristics	PD	iRBD
	Verbal fluency: N=3	Verbal fluency: N=2

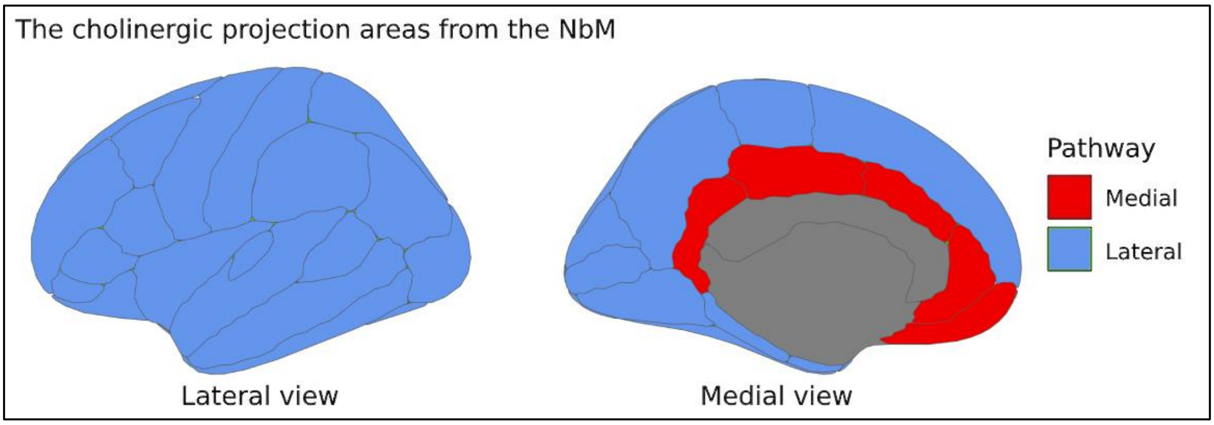

Supplementary Figure 1. The cholinergic projection areas from the NbM. NbM cortical projection areas used for streamlines counts based on the white matter pathways of the NbM described by Selden et al. (1998)[6]. The lateral pathway's projection area (green) includes streamlines from dorsal and lateral frontal cortices, superior, inferior and medial parietal cortices, superior, middle and inferior temporal gyri, inferotemporal cortex, parahippocampal gyrus, occipital cortices, frontoparietal opercular cortices, and insula. The medial pathway's projection area (red) includes streamlines from medial orbitofrontal, cingulate, and retrosplenial cortices. The open-source ggseg package in R was used for the schematic illustration. NbM = Nucleus basalis of Meynert

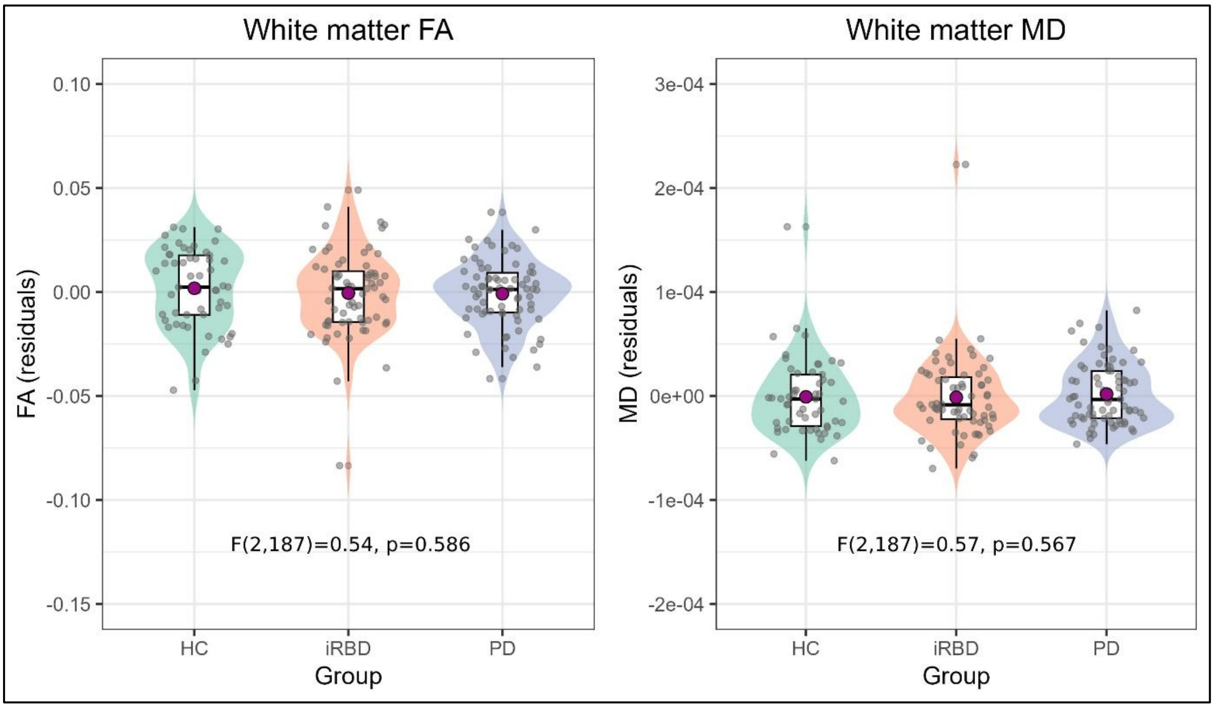

Supplementary Figure 2. Analysis of covariance (n=193) reviled no between-group differences in FA or MD of the whole brain white matter (subtracting the lateral or medial pathways) across the study groups. Purple circles within boxplots represent group means. Individual data points represent individual participants in each group. FA = fractional anisotropy; HC = healthy controls; iRBD = isolated REM-sleep behaviour disorder; MD = mean diffusivity; PD = Parkinson's disease

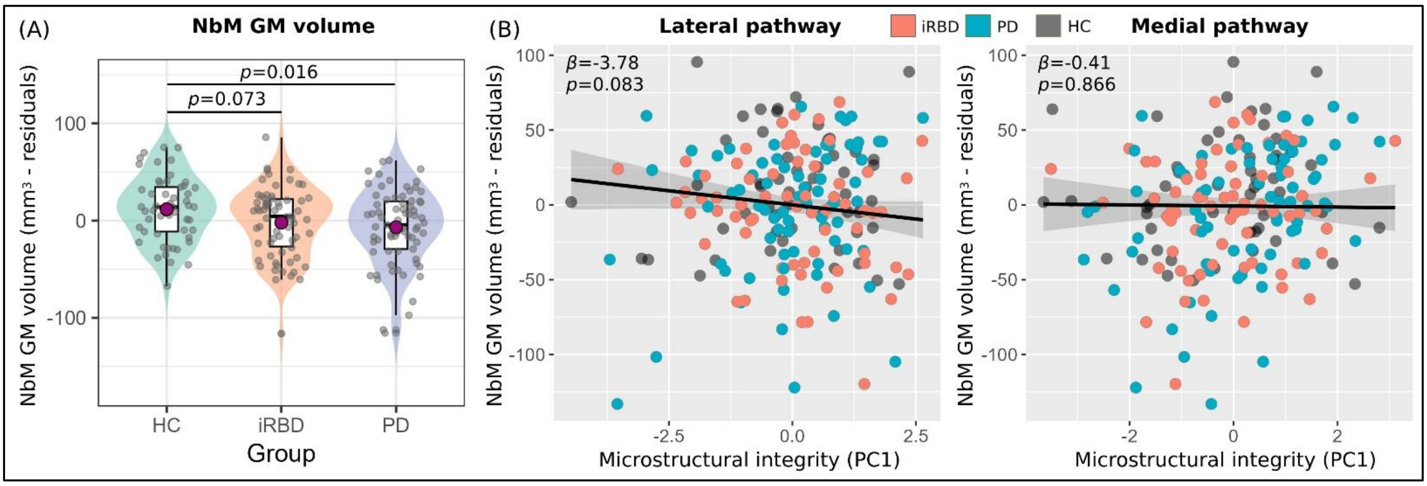

Supplementary Figure 3. (A) Between-group differences in NbM GM volume (based on analysis of covariance, n=192). Individual data points represent individual participants in each group, and purple circles within boxplots represent group means (B) Association between cholinergic pathways microstructural integrity and NbM volume (based on linear regression, n=192). Individual data points represent individual participants in the PD group (cyan) or RBD group (red). FA = fractional anisotropy; GM = grey matter; HC = healthy controls; iRBD = isolated REM-sleep

behaviour disorder; MD = mean diffusivity; NbM = Nucleus basalis of Meynert; PC1 = first principal component; PD = Parkinson's disease

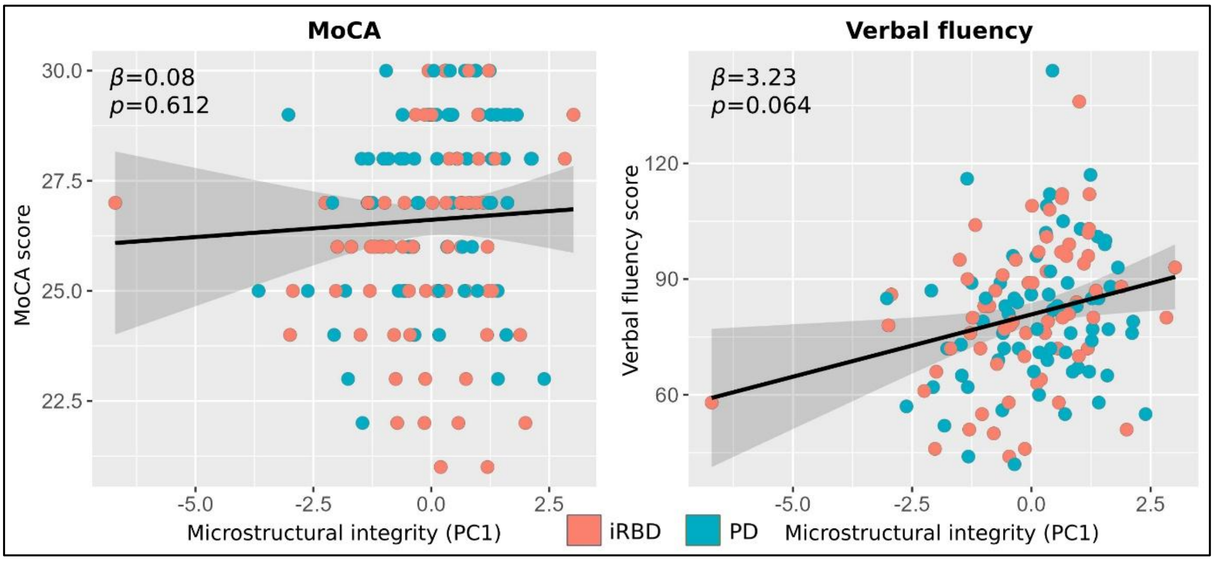

Supplementary Figure 4. Associations between whole brain white matter integrity and baseline MoCA (left panel) and verbal fluency (right panel) scores (based on linear regression, n=140 for MoCA, n=138 for verbal fluency). Individual data points represent individual participants in the PD group (cyan) or RBD group (red). iRBD = isolated REM-sleep behaviour disorder; MoCA = Montreal cognitive assessment; PC1 = first principal component; PD = Parkinson's disease

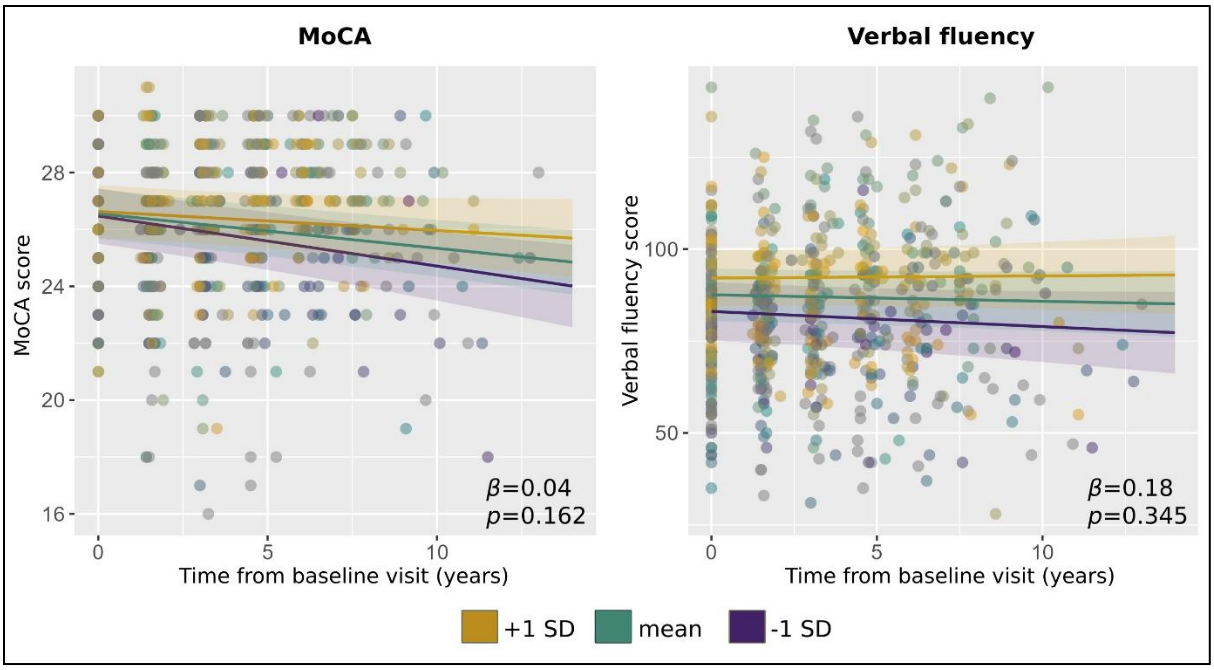

Supplementary Figure 5. Whole brain white matter integrity integrity at baseline and change over time in MoCA score (left panel, non-significant time x baseline microstructural integrity interaction) and verbal fluency score (right panel, non-significant time x baseline microstructural integrity interaction). For visualization purposes of the interaction between cognitive change over time and baseline microstructural integrity, three different slopes representing three levels of baseline microstructural integrity are presented (green = 1 standard deviation (SD) above the mean at baseline, blue=mean at baseline, red = 1 standard deviation below the mean at baseline). The β-values represent the interaction terms' parameter estimates in the models. The plots are based on repeated-measures linear mixed models analyses performed with longitudinal MoCA data points (n=589) and longitudinal verbal fluency data points (n=587) from 139 participants. MoCA = Montreal cognitive assessment; SD = standard deviation

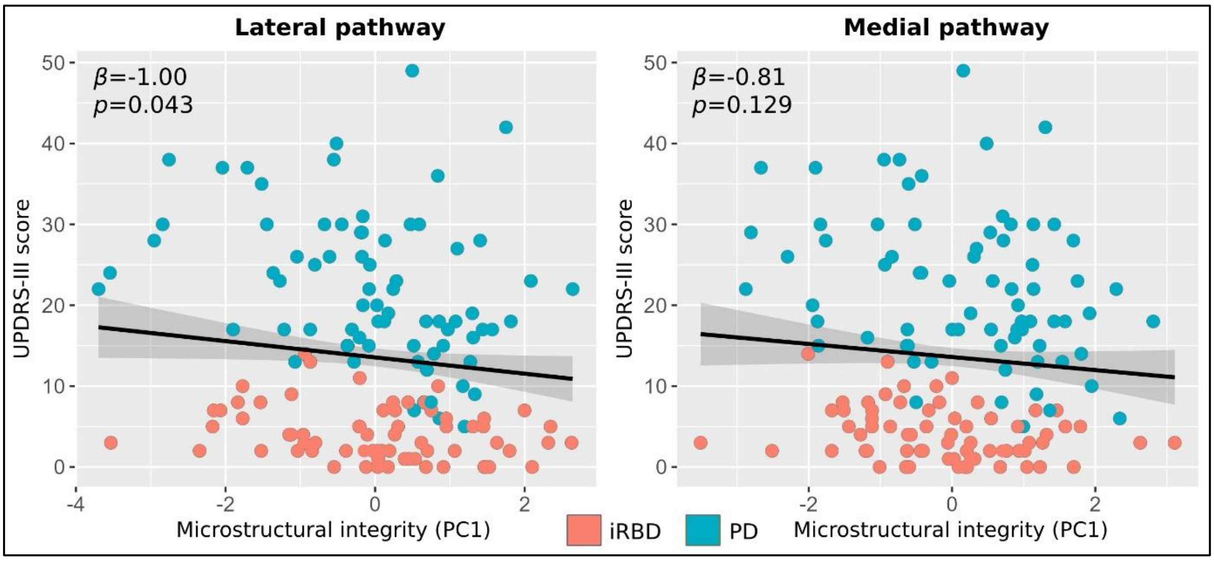

Supplementary Figure 6. Associations between UPDRS-III score and microstructural integrity of the lateral (*β*=-1.00 [95% CI –1.98, -0.03], *p*=0.0430 and medial (*β*=-0.81 [95% CI -1.85, 0.24], *p*=0.129) pathways, based on linear regression analysis (n=139). Note that despite a statistically significant relationship for the lateral pathway, the effect sizes for pathways are very modest (calculated for every 1 SD increase in microstructural integrity). No group-based interaction was demonstrated for either of the pathways (*p*>0.05). Individual data points represent individual participants in the PD group (cyan) or RBD group (red). iRBD = isolated REM-sleep behaviour disorder; PC1 = first principal component; PD = Parkinson's disease; UPDRS-III = Unified Parkinson's Disease Rating Scale - part III

1. Glasser MF, Sotiropoulos SN, Wilson JA, et al. The minimal preprocessing pipelines for the Human Connectome Project. *Neuroimage*. 2013;80:105-124.

doi:10.1016/j.neuroimage.2013.04.127

2. Jenkinson M, Beckmann CF, Behrens TEJ, Woolrich MW, Smith SM. FSL. *Neuroimage*. 2012;62(2):782-790. doi:https://doi.org/10.1016/j.neuroimage.2011.09.015

3. Andersson JLR, Jenkinson M, Andersson JLR. Non-linear optimisation FMRIB Technial Report TR 07 JA 1. In: ; 2007. https://api.semanticscholar.org/CorpusID:12921662

4. Behrens TEJ, Berg HJ, Jbabdi S, Rushworth MFS, Woolrich MW. Probabilistic diffusion tractography with multiple fibre orientations: What can we gain? *Neuroimage*. 2007;34(1):144-155. doi:https://doi.org/10.1016/j.neuroimage.2006.09.018

5. Good CD, Johnsrude IS, Ashburner J, Henson RNA, Friston KJ, Frackowiak RSJ. A Voxel-Based Morphometric Study of Ageing in 465 Normal Adult Human Brains. *Neuroimage*. 2001;14(1):21-36. doi:https://doi.org/10.1006/nimg.2001.0786

6. Selden NR, Gitelman DR, Salamon-Murayama N, Parrish TB, Mesulam MM. Trajectories of cholinergic pathways within the cerebral hemispheres of the human brain. *Brain*. 1998;121 (Pt 1:2249-2257. doi:10.1093/brain/121.12.2249
